# The Therapeutic Action of Quinine

**Published:** 1880-04-20

**Authors:** J. W. Compton

**Affiliations:** Indiana


### Southern Medical Record.

EDITORS:

T. S. POWELL, M.D. W. T. GOLDSMITH, M.D. R. C. WORD, M.D.

*R. C. WORD, M.D., Managing Editor.*

☞All Communications and Letters on Business connected with the RECORD must be addressed to the Managing Editor.

VOL. X. ATLANTA, GA., APRIL 20, 1880. No. 4.

### ORIGINAL AND SELECTED ARTICLES.

### *THE THERAPEUTIC ACTION OF QUININE.*

BY J. W. COMPTON, M.D., OF IND.

The therapeutic value of quinine does not consist alone in its most thoroughly established curative effects in intermittents, or in other words, in its antiperiodic properties.

The good offices of quinine in the cure of intermittent and remittent disease is an established fact as familiar to the public as to the medical profession.

Its value as a remedial agent may be classed under the following heads, as their definition will invite attention to the manifold therapeutic advantages that may be derived from a proper administration of this valuable remedy, viz :

Anti-miasmatic, Anti-septic, Anti-phlogistic, Anti-pyretic, Anti-neuralgic, Prophylactic, and probably Oxytoxic.

A few words only need be said in reference to a fact so thoroughly understood as is the anti-miasmatic powers of quinine.

It is equally familiar to all who have passed any considerable time in paludal districts, that a poison is generated and that this poison has received the name of malaria; and that this malaria enters the human system, inducing certain morbid processes which manifest themselves in the great variety of symptoms peculiar to ague, and the numerous train of phenomena embraced in the collateral intermittents.

This malarial poison is generated at a season when vegetation is most luxuriant, most abundant, and approaching the state of fermentation and decomposition.

This fermentation and putrescent decomposition of vegetable matter, when exposed to the high temperature of a burning sun, develops and multiplies vast quantities of malarial germs, or low organisms; these are carried everywhere in the air, enter the human body through the atmosphere we breathe, the food we eat, the water we drink, and find-ind a congenial habitation, a proper temperature in the human secretions, develop and multiply in the blood and through it is carried to every part reached by the circulation. The system becomes thoroughly saturated, the peculiar train of symptoms ensues and culminates in a morbid condition of poisoning known as malarial disease in its multitudinous forms. The malarial or miasmatic poison producing this class of diseases being generated at a season of the year which favors both fermentative and putrescent action in vegetation and stagnant water, the anti-septic properties of quinine are strongly indicated and are capable of easy demonstration in practice. Quinine is the remedy, par excellence, in nearly all forms of miasmatic diseases. It will, for a long time, preserve in a fresh state, flesh, meal, milk, butter, urine, albumen, etc., and will check alcoholic fermentation in honey or preparations containing sugar, by killing the microscopic organisms that are the immediate cause of these changes.

It exerts a poisonous and fatal effect on all infusorial life. When these have produced the poison and it has entered the human system, quinine will arrest their further action when it comes in contact with them in the stomach or in the blood.

The anti-phlogistic and anti-pyretic properties of quinine may be considered at the same time. Many failures to obtain satisfactory results from this indispensable medicine are caused by a want of considering how widely different is the physiological action of a small dose from that of a large one. There is a great want of this kind of discrimination shown; indeed, it is unscientific to speak of quinine having such and such actions without stating or qualifying the amount of dose necessary to produce a specified action, for corresponding to the size of the dose we will have actions not only altogether different in degree, but even antagonistic and opposite in character.

These different actions of quinine may be illustrated by comparing malarial diseases, or the germs which cause them, if you choose, to a field of tall grain, and the remedy to the action of wind currents on the grain. A gentle breeze blowing across the field will cause the grain to bend and lean in the direction the mild force of the wind inclines it. When the wind ceases it will rise up again.

Small doses of quinine will have a depressing or mildly antagonistic action on the disease, or in other words, will subdue the activity of some of the germs; but withdraw the remedy, and the renewed activity will be manifested by a return of all the previous symptoms.

A strong gale would break down some of the grain, and it would never rise again. A relatively increased dose of quinine would so subdue the disease that some of the symptoms would never return. The extra large dose, like the fierce tornado that sweeps and breaks down everything in its track, would not only destroy the cause of the disease, but would so antagonize the morbid manifestations as to leave the disease completely prostrated, never to rise again until a new supply of the cause had again entered the system. It has been established, both clinically and physiologically, that quinine in large doses has the undoubted power of lowering animal temperature, that it outranks every other article of the materia medica in reducing high grades of fever or exalted temperature in the human body.

It is a universally acknowledged fact that quinine in small doses stands pre-eminent as a tonic and stimulant, that persons who have long accustomed themselves to take regular stimulant doses, on suddenly withdrawing the drug feel the loss or want of it, not to the same degree, but similar to that felt by the toper on the withdrawal of his accustomed stimulant.

If quinine, then, in large or small doses produced similar effects, such different results as clinical experience have demonstrated would be impossible. The stimulant doses revive failing activity, while the large doses depress exalted activity, and it is only when the practitioner considers the action of this remedy in accordance with these principles that its diverse actions are properly understood.

Dr. C. Liebermeister claims that by a very large number of experiments he has demonstrated its power of lessening fever heat. He asserts that he has given some ten thousand doses of quinine as an antipyretic and has almost unbounded confidence in it. He insists that from twenty to forty grains must be given within the hour, and not repeated oftener than once in twenty-four hours.

In regard to its anti-neuralgic powers, it is only necessary to state that, should the neuralgia depend on malarial causes, the indication for its employment is quite manifest. Should the neuralgia, however, be of a non-malarial intermittent character, the influence of the remedy upon the nervous system will enable it to be a valuable curative agent in controlling affections of this character.

It is not my purpose to enter into a discussion of the great variety of diseases in which this drug may be employed with great advantage. I cannot, however, leave the subject without calling especial attention to its action in pernicious fevers and malignant malarial poisoning, without a few words of precaution as to the mode of its administration. In these diseases the storm power of the remedy is imperatively demanded. The patient should be thoroughly cinchonized immediately after the first paroxysm. At least forty grains of the remedy should be administered during the first twenty-four hours, and twenty-five grains during the second; in very severe cases much larger doses than even these may be necessary.

The prophylactic value of quinine has been thoroughly tested in all portions of the inhabited world. Small daily doses administered to persons exposed to malarial influences have been found to be quite as reliable in preventing malarial diseases as it was efficacious in curing them. Wherever the prophylactic powers of quinine have been tested even on the largest scale, in connection with the army and naval service, the testimony in its favor has been unanimous.

Dr. J. B. Hamilton (*Indian Medical Gazette*) reports a battery of one hundred and thirty-five men quartered at Jubbulpore, East India, in the same barracks with an infantry regiment. Each of the artillerists received three grains of quinine every other day, to the infantry none was given. The result was that three hundred out of the five hundred men of the regiment were sick at one time with malarial disease, while at no period was more than four per cent. of the battery affected. The dose of quinine as a prophylactic in very malarial climates should be from three to five grains daily.

In regard to the oxytocic properties of quinine, I should feel inclined to pass them by without comment, were it not for the diverse and opposite opinions which prevail in the ranks of the medical profession; and for the apprehension I entertain, that many pregnant women, suffering from malarial disease, would have their health seriously impaired, even die from witholding this indispensable remedy in that class of diseases, to the attacks of which they are quite liable as other persons, and in whose cases the remedy is as imperatively demanded.

From Dr. H. C. Wood we learn that, in 1871, Dr. Monteverdi announced that quinine is a uterine stimulant, causing at times in the gravid womb contractions sufficiently violent to induce abortion, and when given during labor, intensifying greatly the uterine pains, and after labor causing rapid expulsion of the placenta and arresting uterine hemorrhage; affirming further that in amenorrhea or in menorrhagia from uterine inertia its action is no less marked.

Dr. Jos. J. West says that many regard the use of quinine as dangerous, even criminal in any disease in pregnant women. The belief of these persons is that this substance exercises a direct influence upon the uterus, causing powerful contractions and expulsion of the fœtus. And to support this notion they are ready to bring forward innumerable instances of abortion after its use, of cases of sudden suppression relieved by the prompt use of the same remedy. He then goes on to say that those abortions, etc., were due to the intermittent fever and not to the drug. In this latter opinion my experience would lead me to arrive at the same conclusions. The difficulty of proving a negative in these cases is apparent.

Opposed, however, to the theory that ascribes abortifacient properties to quinine, is the fact that in malarial districts it has been an indispensable remedy for a great length of time in miasmatic diseases, being used indiscriminately without any such property being attributed to it until a comparatively recent date.

Many authorities could be named who gave the remedy to hundreds of pregnant women, without, in the slightest perceptible degree, producing uterine contractions. Altogether it is a reasonable conclusion that instead of quinine originating and producing the abortions charged to its account, that malarial disease causes the contractions in a way that, I think, is easy to explain and that the drug is the most reliable remedy we possess to prevent the abortions by arresting the disease.

The tendency of intermittents to disturb the nerve centers, to produce a shock of the system, is well understood. They also produce serious congestions of internal organs in which the uterus receives its full share of engorgement, a determination of blood to the womb sufficient to stimulate it to contraction, the uterine blood-vessels become engorged and a collection of serum and blood between the chorion and amnion will detach the placenta and the extravasation of blood from the uterine sinuses will force it from its connection with the uterus, hemorrhage takes place and abortion follows. Another fruitful source of abortion is the excessive vomiting so frequently the result of gastric trouble in malarial disease. The instances in which physicians have been called to cases of threatened abortion and premature labor succeeding malarial attacks, where no quinine had been taken, are frequent and numerous. They find the labor so far advanced, with contractions, hemorrhage, or dilatation with protrusion of the membranous sack, as to preclude the success of preventives to arrest its further progress and abortion or delivery inevitable; and all this for the want of the timely use of quinine to control the tendency of malarial attacks to produce shock and congestions.

In conclusion, permit me to counsel all who may have this class of disease to contend with, that they will find malarial disease a much more fruitful danger to pregnant women than is the remedy necessary to break up an intermittent. It is strongly asserted by many obstetricians, and may yet be borne out by clinical experience, that contractions of the uterus are increased by quinine. This may be true, that by virtue of its tonic stimulant properties transmitted through the nerves to the muscular system, that quinine may be utilized in cases of inertia of the uterus, manifested by a relaxed condition of the system and a general loss of tone and muscular power, where the pains have ceased from fatigue during the latter stages of labor, that tonic doses may impart sufficient energy to enable labor to be completed without other interference; yet, as a remedy, it could not be relied upon to originate pains or to materially hasten delivery on other physiological grounds.

